# Phylogeography of the sand dollar genus *Encope*: implications regarding the Central American Isthmus and rates of molecular evolution

**DOI:** 10.1038/s41598-017-11875-w

**Published:** 2017-09-14

**Authors:** Simon E. Coppard, H. A. Lessios

**Affiliations:** 1 Smithsonian Tropical Research Institute, Box 0843–03092 Balboa, Panama; 2 Present Address: Hamilton College, Department of Biology, 198 College Hill Road, Clinton, New York 13323 USA

## Abstract

Vicariant events have been widely used to calibrate rates of molecular evolution, the completion of the Central American Isthmus more extensively than any other. Recent studies have claimed that rather than the generally accepted date of ~3 million years ago (Ma), the Isthmus was effectively complete by the middle Miocene, 13 Ma. We present a fossil calibrated phylogeny of the new world sand dollar genus *Encope*, based on one nuclear and four mitochondrial genes, calibrated with fossils at multiple nodes. Present day distributions of *Encope* are likely the result of multiple range contractions and extinction events. Most species are now endemic to a single region, but one widely distributed species in each ocean is composed of morphotypes previously described as separate species. The most recent separation between eastern Pacific and Caribbean extant clades occurred at 4.90 Ma, indicating that the Isthmus of Panama allowed genetic exchange until the Pliocene. The rate of evolution of mitochondrial genes in *Encope* has been ten times slower than in the closely related genera *Mellita* and *Lanthonia*. This large difference in rates suggests that splits between eastern Pacific and Caribbean biota, dated on the assumption of a “universal” mitochondrial DNA clock are not valid.

## Introduction

The use of vicariant events to calibrate rates of molecular evolution and date phylogenies has a long history, going back to Maxson *et al*.’s^1^ dating of phylogenetic relations in marsupials and frogs, on the basis of continental drift. The vicariant event most extensively used for marine organisms has been the separation of the Pacific and Atlantic Oceans by the Central American Isthmus^2^. Evidence from paleontology, paleoceanography, and sedimentology dated the separation of the eastern Pacific from the Caribbean at ~3 million years ago (Ma)^3–5^. This date provides a minimum estimate of the time that marine organisms in the two oceans have evolved independently from one another. In addition to studies of allopatric speciation^6, 7^, divergence between Atlantic and Pacific organisms has been extensively used to calibrate taxon specific molecular clocks. As of January 2008, 251 studies of molecular evolution relied on such calibrations^2^.

Some authors have challenged the use of vicariance to date evolutionary events^8–10^. A series of recent publications regarding the geological history of the Isthmus of Panama^11–13^ has added to their arguments. Montes *et al*.^13^ came to the conclusion that the Isthmus was closed by the Middle Miocene, 15–13 Ma, though they allowed that “continued Caribbean-Pacific water exchange may have taken place along narrow, shallow, and transient channels that fragmented the Isthmus”. The width, depth, or duration of such channels was not specified. From the point of view of marine organism evolution, it is crucial to know whether these water connections were narrow enough to restrict genetic exchange between marine organisms in the two oceans. The claim of an early Isthmus has also promoted a study that, instead of using vicariance to date phylogenies, used phylogenies to date the vicariant event. Bacon *et al*.^14^ reconstructed the history of the Isthmus by assuming a “universal” rate of divergence of 2% per Ma for mitochondrial DNA (mtDNA) for all marine organisms (see Bacon *et al*.^14^ supporting information, 1.5). Applying this calibration to species pairs of marine organisms presumed to have been separated by the Isthmus, they found genetic splits said to reflect events of vicariance concentrated at 23, 7 and 2 Ma and thus suggested that there was a complex history of land connections. O’Dea *et al*.^5^ pointed out that drawing conclusions on the assumption of a universal divergence rate in mtDNA is likely to lead to erroneous conclusions. They, instead, compiled evidence from 17 molecular phylogenies calibrated with fossils at various nodes; these phylogenies contained 38 sister clades with one member in each ocean. Applying a relaxed clock^15^ to these phylogenies, they determined the date of splitting between sister clades one on each coast of Panama. As one might expect, the results showed that some Atlantic and Pacific separations occurred (or appeared to occur, because of extinctions) in the distant past. However, the most recent split in the 17 fossil-calibrated phylogenies occurred in an echinoderm, the sand dollar genus *Mellita* (now split into *Mellita* and *Lanthonia* ^16^). The estimated time of separation between the Caribbean *M*. *quinquiesperforata* and the Pacific clade of *M*. *notabilis*, calibrated independently of the Isthmus, was 3.21 Ma, suggesting that there were water connections until then^17^.

The validity of assuming a universal rate of molecular evolution in connection with the rise of the Isthmus is in need of corroboration from more taxa with good fossil records, ideally taxa other than mollusks and fishes, which composed the majority of cases presented by O’Dea *et al*.^5^. As a rule, echinoids are a group with a poor fossil record^18, 19^. Most occupy hard bottom habitats and have fragile tests, which fossilize poorly, making species identifications difficult. This disadvantage, however, is greatly mitigated in sand dollars, because their infaunal sandy habitats and their low-volume flat tests are conducive to good preservation. Among sand dollars, members of the neotropical genus *Encope* are the most diverse and best preserved, because their robust tests often remain unbroken^20, 21^. Fossils of *Encope* are present as far back as the Middle Miocene^22^, while the majority of fossils belonging to extant species date back to the Pliocene or Pleistocene. Thus, *Encope* provides the opportunity to construct a molecular phylogeny calibrated at multiple points by fossil constraints, and therefore provide reliable information of the rate of molecular evolution and of the time of separation between clades, including those separated by the Isthmus of Panama. Such a phylogeny is also useful in providing systematic data regarding this genus, in which multiple species have been described on the basis of slight morphological differences.

*Encope* is a strictly New World genus^23^. Species are mostly intertidal or subtidal^24^, though a species from the Galapagos was recorded from a depth of 130 m and another from the Revillagigedos from 82 m^25^. The systematic literature regarding the genus is replete with confusion regarding morphological forms that have been described sometimes as species, others as subspecies, and yet others as varieties^24, 26^. Currently, six extant species of *Encope* are thought to occur on the Atlantic coast of America. *Encope aberrans* and *E*. *michelini* range from North Carolina, around both coasts of Florida and throughout the Gulf of Mexico, to Yucatan. The former is also present in the Bahamas^27^ and at Hispaniola^28^. *Encope emarginata* ranges from South of Yucatan to Uruguay^29^; it has recently been reported from the extreme southeastern coast of South America^30^. *Encope oblonga* and *E*. *subclausa* were established for specimens from Brazil, and *E*. *valenciennesii* for specimens from Martinique^31^. Sixteen nominal extant species or subspecies of *Encope* have been described from the eastern Pacific^26^. *Encope borealis*, *E*. *grandis*, and *E*. *californica* are endemic to the Gulf of California. *Encope arcensis* has also been listed as extant and endemic to the Gulf of California^26^, but this species was established for specimens from the lower Pliocene and has no living representatives. *Encope micropora* extends from the Gulf of California to Callao, Peru^24^, *E*. *micropora fragilis* is recorded from the Pacific coast of Mexico, *E*. *laevis* from Nicaragua, *E*. *micropora tetrapora* from the Galapagos and Panama^24^, *E*. *micropora irregularis* from Costa Rica to Colombia, *E*. *micropora ecuadorensis* from Ecuador, and *E*. *perspectiva* and *E*. *wetmorei* from Mexico to Panama^32^. One species and three subspecies of *Encope* are endemic to island groups of the eastern Pacific. *Encope galapagensis* has been described from the Galapagos^32^, *E*. *perspectiva jonesi* was established for a specimen from San Cristobal Island in the Galapagos^32^, *E*. *micropora insularis* from the Revillagigedos, and *E*. *micropora cocosi* from Isla del Coco^25^.

In the only published effort to establish species relations within *Encope*, Phelan^33^ divided the genus into two subgenera based on test plating of the oral surface. He placed *E*. *micropora*, *E*. *perspectiva*, *E*. *wetmorei* and *E*. *emarginata* in the subgenus *Encope Echinadesma*, separating them from *E*. *aberrans*, *E*. *michelini* and *E*. *grandis*, which he retained in *Encope Encope*. This division was accepted by Kroh & Mooi^26^. The taxonomic position of *Mellitella (Encope) stokesii* is uncertain. Mortensen^24^ and Cooke^34^ considered *Mellitella* to be a subgenus of *Encope*, whereas Mooi^35^ and Smith^36^ considered it as a separate genus. *Mellitella stokesii* occurs in the eastern Pacific from Mexico to Ecuador and is also recorded from the Galapagos Islands^37^.

*Encope michelini* and *E*. *aberrans* have pluteus larvae that can develop to the eight-arm stage without feeding. If food is available, they metamorphose in 9–11 days or 5–7 days respectively^38, 39^. There is no information regarding the development of all other species of *Encope*, but most likely they have planktotrophic larvae.

In this study we combine mitochondrial and nuclear DNA sequences to reconstruct the phylogeny of *Encope*, and address the question of how long ago Caribbean and eastern Pacific populations ceased to be genetically connected. We also use the molecular data to assess the validity of *Mellitella* as a separate genus and ask whether species of *Encope* recognized by morphology are valid. Finally, we compare rates of evolution of genes sampled in common in *Encope*, *Mellita* and *Lanthonia*, genera in the same family, to determine the extent of variation in substitution rates among closely related taxa of sand dollars.

## Results

### Phylogeny

**Figure 1:**
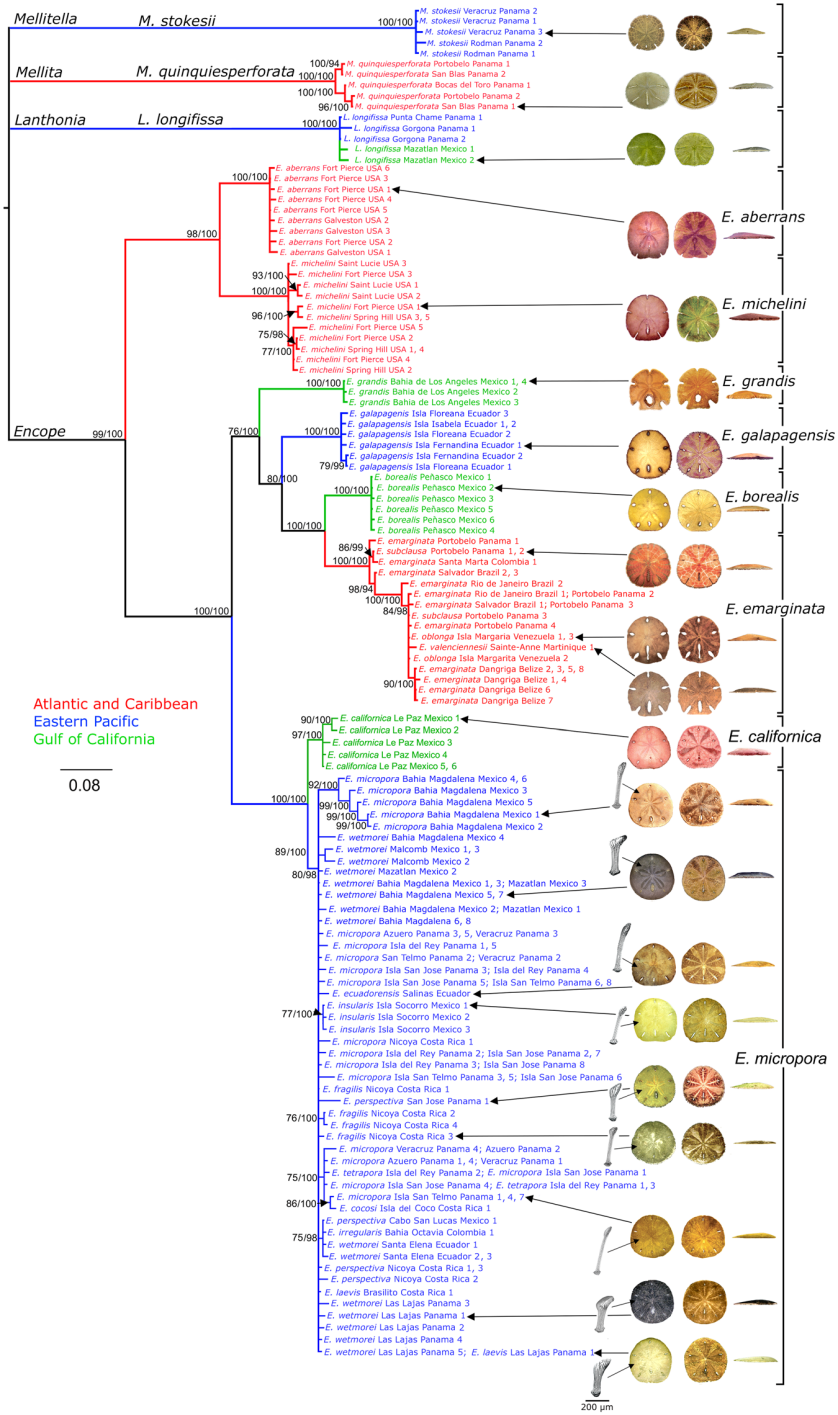
Phylogeny of *Encope* and the other genera of Mellitidae based on concatenated COI, ATPase-6, ATPase-8, 16S and 28S data reconstructed with MrBayes and RAxML and rooted on *L*. *sexiesperforata*. Clade credibility values >75% of Maximum Likelihood (first number next to node) and >85% of Bayesian (second number) reconstruction are shown. Numbers after locality names indicate individuals with indistinguishable haplotypes, scale bar reflects number of changes per site. Names next to terminal branches indicate the morphology of the specimens; names to the right of the pictures are our interpretation as to species affiliation according to the molecular phylogeny. Colors represent geographic range (red = Atlantic and Caribbean, blue = eastern Pacific, green = Gulf of California, black = unknown). Aboral club-spine structures (lateral view) are shown next to subclades in *E*. *micropora*.

**Figure 2:**
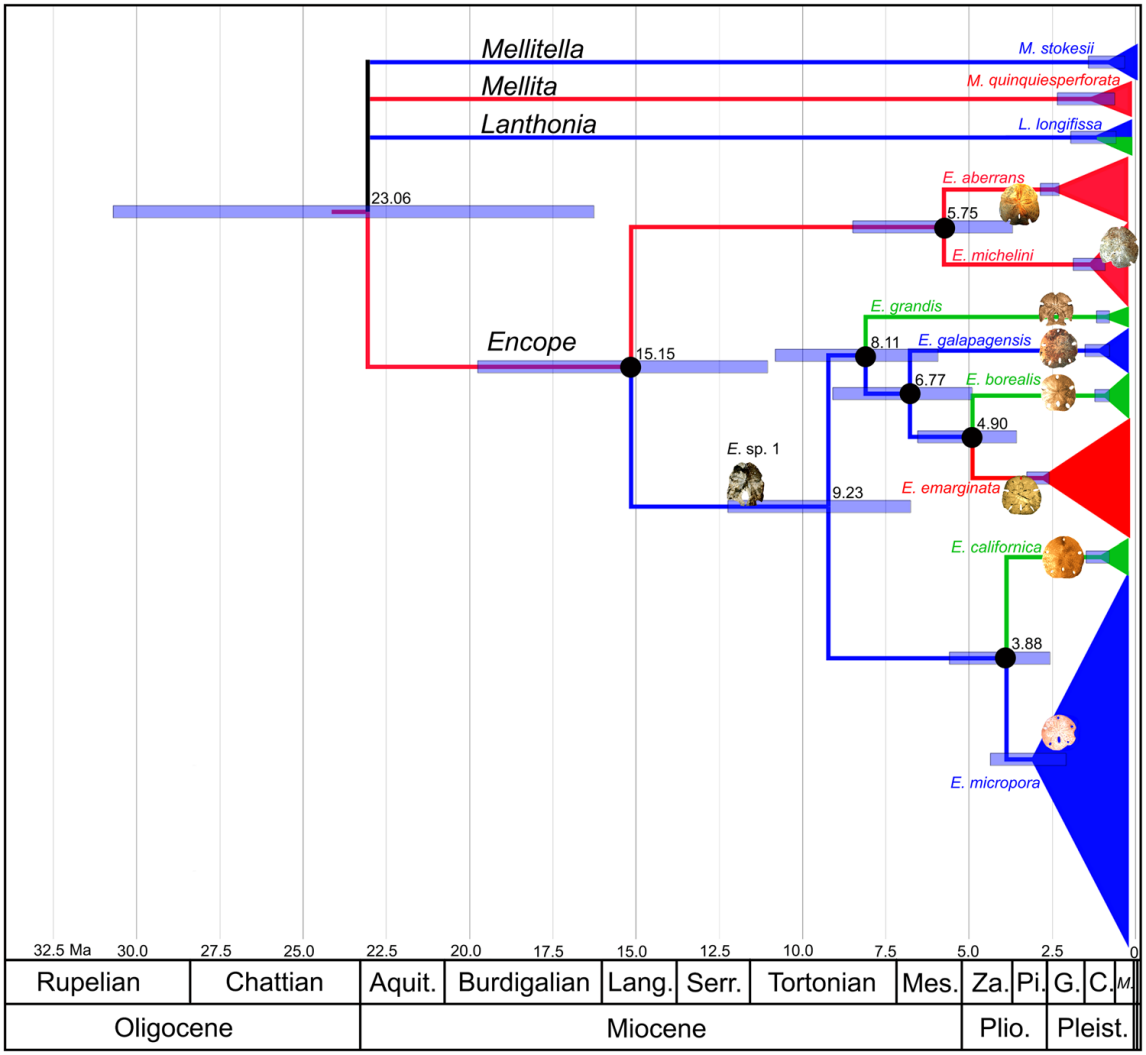
Bayesian estimates of median molecular divergence times (dates next to nodes) based on concatenated COI, ATPase-6, ATPase-8, 16S, and 28S data, as derived from analysis using BEAST and calibrated using the fossil record. Black dots indicate temporally constrained nodes. Images of fossils indicate the oldest known occurrence of each constrained species. Bars indicate 95% Highest Posterior Density (HPD) limits. The fossil designated *E*. sp. 1 is the undescribed species from the Gatun Formation mentioned in the text. Ages of stages and epoch series are based on International Commission on Stratigraphy stratigraphic chart^90^ (Aquit. = Aquitanian, Lang. = Langhian, Serr. = Serravallian, Mes. = Messinian, Za. = Zanclean, Pi. = Piacenzian, Plio. = Pliocene, G. = Gelasian, C. = Calabrian, *M*. = *Middle*, Pleist. = Pleistocene). Error bars represent 95% Highest Posterior Density (HPD) intervals.

Bayesian inference (BI) and Maximum likelihood (ML) analyses of the concatenated data of five genes of *Encope*, *Mellita*, *Lanthonia*, and *Mellitella*, rooted on *Leodia*, produced congruent phylogenies, with only slight differences in weakly supported subclades within species (Fig. 1). Cross validation of the node dates through re-estimation by removing one fossil offset at a time indicated the estimates to be robust. All median dates thus estimated fell well within the Highest Posterior Density (HPD) ranges of the run based on all eight fossil constraints (Table S1). Nominal genera resulted as different phylogenetic entities, separated from each other 26-16 Ma (Fig. 2). *Mellitella stokesii* was similarly distinct, justifying Mooi’s^35^ removal of this species from *Encope*. Phelan’s^33^ subdivision of the genus *Encope* was not supported by the molecular phylogeny, because *E*. *grandis* was not related to *E*. *aberrans* and *E*. *michelini* as he supposed, but shared a clade with *E*. *galapagensis*, *E*. *borealis* and *E*. *emarginata*.

The clade composed of the Atlantic species *Encope aberrans* and *E*. *michelini* was the first to have split from all other extant species of *Encope* (Fig. 1) in the Middle Miocene, 15 Ma (95% HPD: 19.76-11.05 Ma) (Fig. 2). Each of these two sympatric morphospecies was monophyletic, having split from each other approximately 6 Ma (95% HPD: 8.49-3.49 Ma). Thus, the molecular phylogeny did not justify A. Agassiz’s^40^ and Mortensen’s^24^ suggestions that they should be considered as conspecific. The sister clade of *E*. *aberrans* and *E*. *michelini* was split 9 Ma (95% HPD: 12.24-6.75 Ma) into a lineage consisting of the eastern Pacific species *E*. *californica* and *E*. *micropora* and a lineage that included three Pacific species, *E*. *grandis*, *E*. *galapagensis*, and *E*. *borealis*, plus an Atlantic one, *E*. *emarginata*. In the clade that occupies both oceans, *E*. *grandis*, endemic to the Gulf of California, was the first one to split 8 Ma (95% HPD: 10.82-5.93 Ma), whereas *E*. *galapagensis*, endemic to the Galapagos, split from the transisthmian pair nearly 7 Ma (95% HPD: 9.09-4.99 Ma). The transisthmian pair consisted of *E*. *borealis*, now endemic to the Gulf of California, and the western Atlantic *E*. *emarginata*. The BEAST median estimate of this split was 4.90 Ma with a 95% HPD of 6.54-3.57 Ma, indicating that there was exchange of water between the eastern Pacific and the Atlantic until this time. Nested within the Atlantic clade of *E*. *emarginata* are the morphospecies *E*. *subclausa*, *E*. *oblonga* and *E*. *valensciennesii*. Genetic distances between concatenated sequences of *E*. *emarginata*, *E*. *subclausa*, and *E*. *oblonga* ranged between 0.28 and 0.35%; mean intra-morph distances ranged between 0.03 and 0.47% (Table S2). Thus, inter-morph variation was no larger than intra-morph variation, suggesting very strongly that these three morphs belong to the same species. The single specimen of *E*. *valensciennesii* we were able to obtain had similarly small distances in COI, ATPase8 and 28S, but values of 3.48–4.04% in ATPase6 and of 1.38% in 16S, which suggest a certain degree of differentiation (Table S1). There were well-supported subclades within *E*. *emarginata sensu lato*, but they did not correspond to morphological variation, nor did they conform to geographical structure.

The second lineage in the eastern Pacific contained two clades. The genetic distances in all genes between *Encope californica* and the large clade we have designated as *E*. *micropora* was <1.89% (Table 1), but given that the haplotypes form a monophyletic entity that corresponded with distinctive morphology, we consider it as a separate species. This was not the case in the other Pacific clade. The *E*. *micropora* clade contained specimens with a broad range of morphological variation, revealing high levels of plasticity in test and spine structures with no apparent phylogenetic structure. Such morphotypes have been described as separate species or subspecies, but the genetic data contain no compelling information for their separation.

**Table 1:** Maximum composite likelihood distances [1] between clades within *Encope*, *Mellita*, and *Lanthonia* [2] shown in Fig. 2 for each gene separately and for the concatenated sequences. Timing of divergence was calculated in BEAST using the concatenated set of genes with a relaxed log-normal clock with calibrations from the fossil record. Sister clades on either side of Panama are shown in bold.

	Age	Concatenated		16S		COI		ATPase8		ATPase6		28S	
	Ma	Dist.	Rate	Dist.	Rate	Dist.	Rate	Dist.	Rate	Dist.	Rate	Dist.	Rate
		%	%/My	%	%/My	%	%/My	%	%/My	%	%/My	%	%/My
*Encope*													
(*E*. *abe*., *E*. *mich*.) from (*E*. *gra*. (*E*. *gal*. (*E*. *bor*. *E*.*ema*.)) (*E*. *cal*., *E*. *micr*.))	15.15	5.10	0.34	4.90	0.32	5.81	0.38	13.77	0.91	9.15	0.60	0.10	0.01
(*E*. *gra*. (*E*.*gal*. (*E*. *bor*., *E*. *ema*.))) from (*E*. *cal*., *E*. *micr*.)	9.23	2.88	0.31	2.57	0.28	2.93	0.32	4.17	0.45	5.87	0.64	0.06	0.01
*E*. *grandis* from (*E*. *gal*. (*E*. *bor*., *E*. *ema*.))	8.11	2.79	0.34	2.59	0.32	2.96	0.36	3.85	0.47	5.75	0.71	0.06	0.01
*E*. *gal*. from (*E*. *bor*., *E*. *ema*.)	6.77	1.98	0.29	1.73	0.26	1.94	0.29	3.90	0.58	3.77	0.56	0.09	0.01
*E*. *aberrans* from *E*. *michelini*	5.75	1.84	0.32	0.97	0.17	5.70	0.99	2.73	0.47	3.97	0.69	0.04	0.01
***E***. ***borealis*** **from** ***E***. ***emarginata***	4.90	1.55	0.32	0.83	0.17	1.11	0.23	4.39	0.90	3.99	0.81	0.13	0.03
*E*. *californica* from *E*. *micropora*	3.88	0.60	0.15	0.75	0.19	0.49	0.13	1.89	0.49	1.06	0.27	0.01	0.00
Median rate			0.32		0.26		0.32		0.49		0.64		0.01
*Lanthonia* and *Mellita*													
(*L*. *gran*. (*L*. sp3 (*L*. *long*. (L. sp4)))) from (*M*. *ten*. (*M*. sp2 (*M*. sp1, *M*. *qui*.) (*M*. sp6, *M*. sp5, *M*. *not*.)))	5.46	33.69	6.17	6.83	5.46	44.93	8.23					0.54	0.10
*M*. *tenuis* from (*M*. sp2 (*M*. sp1, *M*. *qui*.) (*M*. sp6, *M*. sp5, *M*. *not*.))	3.83	17.32	4.52	6.19	3.83	23.94	6.25					0.18	0.05
**(** ***M***. **sp2 (** ***M***. **sp1**, ***M***. ***qui***.**)) from (** ***M***. **sp6**, ***M***. **sp5**, ***M***. ***not***.**)**	3.21	18.45	5.75	6.27	3.21	25.20	7.85					0.11	0.03
*M*. sp2 from (*M*. sp1, *M*. *quinquiesperforata*)	3.21	9.06	2.82	2.57	3.21	15.03	4.68					0.03	0.01
*L*. *grantii* from (*L*. sp3 (*L*. *longifissa* (*L*. sp4))	3.12	8.34	2.67	2.17	3.12	14.06	4.51					0.10	0.03
*L*. sp3 from (*L*. *longifissa*, *L*. sp4)	2.38	3.7	1.59	1.26	2.32	6.7	2.89					0.01	0.00
*M*. sp1 from *M*. *quinquiesperforata*	2.23	6.87	3.08	2.87	2.23	10.22	4.58					0.09	0.04
*M*. sp5 from *M*. *notabilis*	1.88	6.52	3.58	2.43	1.82	10.28	5.65					0.02	0.01
*M*. sp6 from *M*. *notabilis*	1.88	8.51	4.68	2.54	1.82	13.56	7.45					0.01	0.01
*M*. sp5 from *M*. sp6	1.88	6.70	3.68	3.40	1.82	9.32	5.12					0.01	0.01
*L*. *longifissa* from *L*. sp4	1.87	3.10	1.71	0.69	1.81	5.67	3.13					0.09	0.05
Median rate			3.58		2.32		5.12						0.03

1 Maximum Composite Likelihood model calculated with rate variation among sites modeled with a gamma distribution (Tamura *et al*.^81^).

2 Gene sequences for species of *Mellita and Lanthonia* from Coppard *et al*.^17^ *E*. *abe*. = *E*. *aberrans*, *E*. *mich*. = *E*. *michelini*, *E*. *gra*. = *E*. *grandis*, *E*. *gal*. = *E*. *galapagensis*, *E*. *bor*. = *E*. *borealis*, *E*. *ema*. = *E*. *emarginata*, *E*. *cal*. = *E*. *californica*, *E*. *micr*. = *E*. *micropora*, *L*. *gran*. = *L*. *grantii*, *L*. *long*. = *L*. *longifissa*, *M*. *ten*. = *M*. *tenuis*, *M*. *not*.* = M*. *notabilis*, *M*. *qui*. = *M*. *quinquiesperforata*, Ma = Millions of years ago, My = Million years, and Dist. = Distance. For definitions of new species of *Mellita* and *Lanthonia* (*M*. or *L*. sp. 1–6) see Coppard *et al*.^17^.

### Rates of molecular evolution

A striking feature of evolution of *Encope* is the extremely low divergence rate in all four mitochondrial genes. A comparison of rates (genetic distance divided by time) of *Encope*, *Mellita* and *Lanthonia* is presented in Table 1. There were few substitutions in 28S in all genera, even between the deepest clades. In 16S and COI, however, *Encope* mean rate of evolution is an order of magnitude slower than that of *Mellita* and *Lanthonia*. Differences remain in the same direction even when rates in *Encope* are biased to be as the fastest possible (as the ratio of the highest Confidence Limit of distance divided by the youngest 95% HPD point of age) and rates in *Mellita* and *Lanthonia* are biased as the slowest possible (as the ratio of the lowest Confidence Limit of distance divided by the oldest HPD point of age). There is no apparent correlation of divergence rate and time since separation in any of the genera (Spearman Rank Correlation Coefficient: *Encope*: r = 0.429, p = 0.297, *Lanthonia-Mellita*: r = 0.129, p = 0.693).

## Discussion

The phylogeny of *Encope* in characterized by four notable features: (a) A phylogeographic signature composed of six species endemic to a restricted area and two species, one in the eastern Pacific and one in the western Atlantic, with a remarkably wide distribution. (b) Extreme morphological plasticity in the widespread species that does not show geographic structure. (c) An unusually slow rate of molecular evolution. (d) A time of separation between sister clades in the two oceans that pre-dates the final closure of the Central American Isthmus, but postdates the recently proposed ancient blockage of seaways. These features are interconnected to produce a story of extinction and range contraction that may have resulted in the relationships of species as they are seen today.

That the eastern Pacific *Encope borealis*, now endemic to the Gulf of California, was separated from the western Atlantic *E*. *emarginata* 5 Ma, when Central America was a peninsula and the only water connections were close to South America^41–43^ indicates either that *E*. *borealis* has undergone a range contraction since the Pliocene, or that the true sister species of *E*. *emarginata* on the other side of the Isthmus of Panama has become extinct. The multitude of extinct “species” of *Encope* ^24, 34, 44–48^ suggests that extinction in this genus has been high. Given the morphological plasticity of *E*. *micropora* and *E*. *emarginata*, some of these fossil species were probably not biological species. However, that these forms no longer exist is consistent with the notion that a pattern of extinction characterizes the evolution of the genus. For example, the subspecies *Encope macrophora macrophora* from the Late Miocene and *E*. *macrophora tamiamiensis* from the Plio-Pleistocene in south-eastern United States are morphologically very close to *E*. *grandis* in the Gulf of California (they have a thick test and test margin, with five notches rather than enclosed lunules in the ambulacra)^49^. *Encope macrophora macrophora* and *E*. *macrophora tamiamiensis* did not survive the changing environment of the western Atlantic that followed the emergence of the Central American Isthmus^5^. That range contractions also happened through time is suggested by the contrast between the wide distributions of the younger clades (those of *E*. *micropora* and *E*. *emarginata*) and the much more endemic distributions of older species (those of *E*. *aberrans*, *E*. *michelini*, *E*. *grandis*, *E*. *borealis* and *E*. *californica*). If extensive range contractions and extinctions have taken place, present day geographic distributions of *Encope* species do not provide reliable information on which to base speculation about the causes of speciation.

We have proposed that a number of morphospecies actually belong to *Encope micropora* and that others belong to *E*. *emarginata*, because in the molecular phylogeny they did not form monophyletic clades and their genetic distances were minuscule. However, we have also found that all genes used in our reconstruction have evolved very slowly, the mitochondrial ones much slower than those in *Mellita* or *Lanthonia*. One might reasonably ask whether haplotypes of the slowly evolving molecules of true species have not yet sorted out and thus that the apparent intraspecific morphological variation is an artifact of the slow molecular evolution. We cannot completely exclude this possibility, particularly for the clade with *E*. *micropora* morphology from Bahia Magdalena, in which several specimens form their own clade, even though this clade does not contain all specimens from this location. However, with the exception of specimens of *E*. *micropora insularis* from Isla Socorro, there was no structure to support these variants either in morphology (as indicated by the specific and subspecific names) or in geographical location. Subclades did not include all members of a morphospecies; the same subclade included morphologically divergent specimens, such as those of *E*. *micropora micropora*, mixed with *E*. *wetmorei* and *E*. *laevis*. There did, however, appear to be a correspondence between morphology and habitat. Members of *E*. *micropora sensu lato* with flattened aboral club-spines forming a contiguous spine canopy and interambulcral lunules positioned between the posterior petals were only found in the surf zone of high energy beaches; members with bulbous-tipped aboral club spines, resulting in an open spine canopy, with interambulacral lunules positioned behind the posterior petals were found in deeper water, in sheltered bays, or on lower energy beaches. Morphological intermediates between these forms, such as those corresponding to *E*. *perspectiva* and *E*. *micropora irregularis* were also present at Isla San Jose (Panama) Bahia Octavia (Colombia), and Golfo de Nicoya (Costa Rica) suggesting a gradation in morphological characters that may correspond to intermediate habitats. Thus, the variation that has led systematists to designate different species and subspecies in the eastern Pacific, may well correspond to ecophenotypes.

The extremely slow rate of molecular evolution of *Encope* relative to *Mellita* and *Lanthonia*, genera in the same family, was surprising. There were no differences between the genera in substitution rate in 28S, but this nuclear gene has evolved so slowly in both cases, that comparisons are based on the incorporation of a few mutations; comparing their rate is, thus, not meaningful. 16S and COI, on the other hand, diverged a minimum of 10 times more slowly in *Encope*. A comparison to divergence across the Central American Isthmus in other echinoid genera^2^ indicates that differentiation of COI in *Encope*, with a transisthmian distance of 1.11%, is only half as large as it is in *Diadema* ^50^, the previously smallest known transisthmian distance, whereas the same gene in *Mellita* with a transisthmian distance of 25.2% is twice as large as it is in *Lytechinus* ^51^, the previously largest known distance. Because *Diadema* and *Lytechinus* lack a fossil record, we cannot determine whether the differences in divergence between their species on the two sides of the Isthmus are due to different times of separation or different rates of evolution. In the sand dollars, on the other hand, time of separation is based on fossil calibrations. Apparent differences in rates can be caused by the vagaries of coalescence^52, 53^, by miscalibration of phylogenies, or by the application of different models of DNA evolution^54^. However the differences between rates in *Encope* and rates in *Mellita* and *Lanthonia* throughout their respective phylogenetic trees are too great to be entirely due to these factors. In the time interval considered here, stratigraphic errors in deciding the age of the fossils could not be wrong by an order of magnitude. Different models of DNA evolution affect the estimated genetic distances between the most divergent taxa within each genus. The maximum likelihood distances presented here for *Mellita* differ somewhat from those calculated from gene-specific models in Coppard *et al*.^17^, but only by a factor of 0.35 in the largest deviation. Nor could the differences in rates be due to saturation of the sequences or some other factor that causes them to depend on time^54^, because the time scales in all genera are similar, and because the trend, if any, in the data is that rates of substitution are higher between the most divergent clades. So what could be the cause of such large differences between closely related genera?

A number of hypotheses have attempted to explain deviations from the molecular clock^55–61^. Unfortunately, we do not know the biology of sand dollars in sufficient detail to distinguish how well each of the possibilities apply to *Encope*, except by excluding some of the alternatives. It is unlikely that differential selection can account for the differences between *Encope* and *Mellita* or *Lanthonia* in the evolution of the same genes. Although mtDNA is under strong selection, most of the variation between species in the three genera is in codon positions that do not alter amino acid composition. Given that the differences are consistent between genes, a demographic factor, affecting all genetic markers, is a more likely explanation. According to the nearly neutral theory Ohta’s^62^ or the theory of coalescence^63^, (though not according to Kimura’s^64^ neutral theory), differences in substitution rate depend on effective population size. In our experience, however, species of *Encope* are much more difficult to encounter than species of *Mellita*, so differences caused by effective population size would be in the opposite direction to the one observed. Similarly, there is no reason to assume that mutation rate is lower in *Encope* by an order of magnitude. We tentatively suggest that differences in body size^55, 57, 65^, with its correlated factors, generation time^66^, metabolic rate^55^ and longevity^58^, may be the most likely explanation for the ten-fold slow-down of mtDNA evolution in *Encope*. Whereas species of *Mellita* and *Lanthonia* have thin tests that rarely exceed 10 cm in diameter, *Encope* lays down a heavy, thick, internally supported test that in some of its species can easily reach 15 cm. The size at which species of *Encope* reach sexual maturity is not known, but Mortensen^24^ remarked that a 3.8 cm specimen of *E*. *emarginata* had not yet developed genital pores. In *Mellita* a 3.8 cm specimen is fully mature. It is, therefore, easy to imagine that generation time in *Encope* is longer than in *Mellita*. Whatever the cause of the differences in mtDNA evolutionary rate in the three mellitids may be, the data presented here strongly illustrate the dangers of applying an assumption of a universal mtDNA clock in obtaining dates of completion for the Isthmus of Panama^14^, or any other event.

That the geminate species of *Encope* were separated 4.9 Ma (95% HPD: 6.54-3.75 Ma) would have been expected and unremarkable before the publication of the articles claiming that only narrow channels connected the eastern Pacific and the Caribbean as of 13 Ma^11–13^, and various claims they generated, including proposals that even the causes of the Great Faunal Interchange^67^ of land organisms crossing between continents need to be re-evaluated^13, 14, 68–71^. The 4.9 Ma date does not coincide with any of the vicariant events suggested by Bacon *et al*.^14^. O’Dea *et al*.^5^ have summarized the geological, paleontological, paleoceanographic and genetic data that show that this claim is not likely to be correct. The molecular data from *Encope* add another piece of evidence that genetic (and thus water) connections between the oceans were strong until the Pliocene.

## Materials and Methods

**Figure 3:**
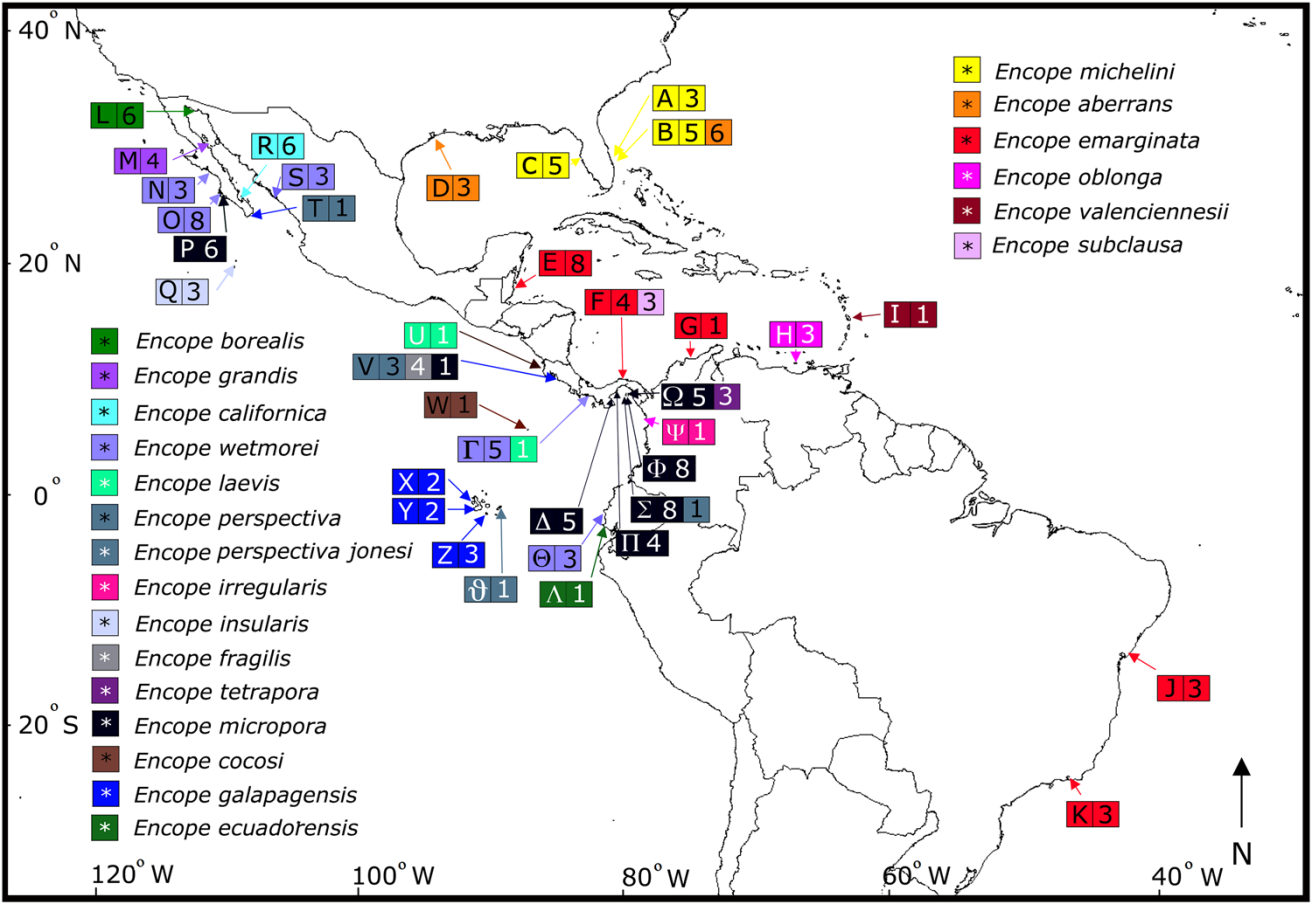
Collection localities of *Encope* used in this study. The letter refers to the locality, the number to the sample size, and the color to the morphospecies. A: Saint Lucie, Florida Fort Pierce, Florida, USA (27.5357°N, 80.3089°W); B: Jensen Beach, Fort Pierce, Florida, USA (27.2680°N, 80.1383°W); C: Spring Hill, Florida, USA (28.4397°N, 82.7709°W); D: Galveston, Texas (29.2994°N, 94.7658°W); E: Pelican Beach, Dangriga, Belize (16.9753°N, 88.2225°W); F: Playa La Angosta, Portobelo, Panama (9.5500°N, 79.6500°W); G: Santa Marta, Colombia (11.2592°N, 74.2050°W); H: Isla Margarita, Venezuela (11.0557°N, 64.1931°W); I: Sainte-Anne, Martinique (14.4397°N, 60.8828°W); J: Ribeira, Salvador, Brazil (12.5252°N, 37.5437°W); K: Bahia Sepetiba, Rio de Janeiro, Brazil (22.9710°N, 43.8772°W); L: Puerto Peñasco, Mexico (31.3143°N, 113.5540°W); M: Bahia de Los Angeles, Mexico (28.9492°N, 113.5576°W); N: Malcomb, Baja California Sur, Mexico (26.7126°N, 113.2670°W); O: Sand Dunes, Bahia Magdalena, Mexico (24.6815°N, 112.1307°W); P: Curvo, Bahia Magdalena, Mexico (24.4518°N, 111.6094°W); Q: Bahia Lucio Gallardo Pavon, Isla Socorro, Islas Revillagigedo, Mexico (18.8379°N, 111.0160°W); R: El Mogote, La Paz, Mexico (24.1568°N, 110.3471°W); S: Isla de la Piedra, Mazatlan (23.1807°N, 106.3926°W); T: Cabo San Lucas, Mexico (22.8694°N, 109.9166°W); U: Brasilito, Costa Rica (10.4084°N, 85.8001°W); V: Golfo de Nicoya, Costa Rica (9.7286°N, 84.8150°W); W: Bahia Wafer, Isla del Coco, Costa Rica (5.5798°N, 87.0687°W); X: Isla Fernandina, Galapagos, Ecuador (0.4532°N, 91.3326°W); Y: Isla Isabela, Galapagos, Ecuador (1.0063°N, 91.0771°W); Z: Isla Floreana, Galapagos, Ecuador (1.2236°N, 90.4379°W); ϑ: Isla San Cristobal, Galapagos, Ecuador (0.9080°N, 89.5568°W); Γ: Playa Las Lajas, Chiriquí, Panama (8.1618°N, 81.8598°W); Δ: Playa Uverito, Azuero Peninsula, Panama (7.7720°N, 80.1744°W); Θ: Punta Blanca, Santa Elena, Ecuador (2.1517°N, 80.7905°W); Λ: Salinas, La Libertad, Ecuador (2.2119°N, 80.9779°W); Π: Playa Venado, Veracruz, Panama (8.8898°N, 79.6007°W); Σ: Isla San Jose, Las Perlas, Panama (8.2514°N, 79.1013°W); Φ: Isla San Telmo Perlas, Panama (8.2746°N, 78.8516°W); Ψ: Bahia Octavia, Colombia (6.8423°N, 77.6223°W); Ω: Isla del Rey, Las Perlas, Panama (8.2792°N, 78.9419°W). The outline of this map was composited from three downloaded files from d-maps.com (http://d-maps.com/carte.php?num_car=1405&lang=en, http://d-maps.com/carte.php?num_car=1389& lang=en and http://d-maps.com/carte.php?num_car=2313&lang=en) and modified in Affinity Photo V. 1.5.2.

All described extant morphospecies of *Encope*, were either collected or borrowed from museums (Fig. 3). Morphological characters of the tests, spines and pedicellariae of each specimen were matched with the original species or sub-species descriptions. Additionally, specimens of *Leodia sexiesperforata*, *Mellita quinquiesperforata*, and *Lanthonia longifissa* were sequenced as outgroups. *Mellitella stokesii* was also sequenced to resolve its phylogenetic position. Muscle from the Aristotle’s lantern of each specimen was preserved in 95% Ethanol for DNA extraction.

### DNA extraction, sequencing and alignment

Genomic DNA was extracted from 150 *Encope*, from four *Leodia sexiesperforata*, and from five each *Mellitella stokesii*, *Mellita quinquiesperforata* and *Lanthonia longifissa* using a DNeasy tissue kit (Qiagen)®. No intact DNA could be extracted from the type specimen of *E*. *perspectiva jonesi*, which may have been fixed in formalin. A 5000 bp fragment of mtDNA was initially sequenced in three species of *Encope* (two Atlantic and one eastern Pacific) and *M*. *stokesii* (eastern Pacific) using 16Sech150FL 5′AGAGACTGGTATGAATGGCAAGAC and NAD3echR 5′ GATTTTAGYGGRTCAAAKCCACAYTCGTA primers. 16S rDNA, cytochrome *c* oxidase subunit I (COI), and the Lysine-tRNA, ATPase-6 and ATPase-8 regions were selected based on the presence of conserved priming regions in all four species. Two overlapping fragments of COI were amplified. The 5′ region of the gene using the primers 5′TTTCAACWAATCAYAARGAC and 5′GGTTCTCGCTTYCCDGA, and the 3′ region using 5′ GCTTTCCTCCTGCTCCTATC and 5′GCATCTGGGTAGTCTGAGTATCGC. The Lysine-tRNA, ATPase-6 and ATPase-8 regions were amplified using primers 5′-GTGAAGGCAGATAACTACTGT and 5′-TCTACAAGGTGGTATGGGTG; a fragment of the 16S rDNA was amplified using primers 5′-CGCCTGTTTACCAAAACA and 5′TCGTAGATAGAAACTGACCTG. The same amplification conditions were employed for all mtDNA regions. They consisted of PCR reactions containing 0.2–0.5 µl of extracted genomic DNA (approximately 10–15 ng), 12.0 µl of nuclease free H_2_0 (adjusted to 12.3 µl when using less genomic DNA), 5.0 µl GoTaq® Flexi Buffer (5x), 2.5 µl MgCl_2_ (25 mM), 2.5 µl dNTPs (8 mM), 1.25 µl (10 mM) of each of the forward and reverse primers, and 0.6 units of Flexi-GoTaq® polymerase (Promega)®. The mixture was cycled 39 times at 96 °C for 10 seconds, 94 °C for 30 s, 50 °C for 45 s, 72 °C for 1 minute, with a final extension at 72 °C for 5 minutes. The 5′ end of nuclear 28 S rDNA was amplified using a HotStartTaq PCR amplification kit (Qiagen)® with the primers and protocol of Stockley *et al*. ^72^. To avoid heterozygous polymorphisms, PCR products of 28 S were cloned using Promega pGEM-T Easy kits®. One randomly chosen clone was cycle-amplified from each specimen using Promega M13 and M13R primers before being sequenced in one direction using the M13 primer. After purification in Sephadex columns, amplification with the same primers, and labeling with Applied Biosystems (ABI) Prism BigDye Terminators, nucleotides were sequenced in both directions in an ABI 3130 XL automatic sequencer. Alignments were performed in MacClade^73^.

After trimming sequence ends of ambiguous bases and overlapping segments, there were 1296 base pairs (bp) of COI, 168 bp of ATPase-8, 687 bp of ATPase-6, 568 bp of 16S and 1137 bp of 28S rDNA, a total of 3856 bp of DNA sequence for each specimen.

### Phylogenetic analyses

Unique haplotypes of each DNA region were subjected to analysis in jModeltest v. 2.1.1^74^ to determine the best model of molecular evolution based on the AIC criterion^75^. The algorithm suggested as the best model for 16S HKY + G^76^ (*α* = 0.0240), for COI TIM3 + I + G^74^ (I = 0.7030, *α* = 1.7370), for ATPase-6 TrN + I + G^74^ (I = 0.6500, *α* = 1.919), for ATPase-8 HKY + G^76^ (*α* = 0.2100), and for 28 S “001120 + I + F”, a model with unequal base frequencies, five rates of base change, no site heterogeneity, and a proportion of 0.884 of invariable sites^74^. These DNA regions were then concatenated, outgroups were added, and they were subjected to phylogenetic analyses with the appropriate model for each gene partition.

Bayesian phylogenetic analysis was carried out with MrBayes v. 3.2^77^ in partitioned analyses applying the best model to each DNA region but allowing the program to estimate values, a heating parameter T of 0.15 and Dirichlet priors for rates and nucleotide frequencies, in 3 × 10^8^ steps, sampling every 3000^th^ tree from two runs. Convergence was assessed as having been reached when the average standard deviation of split frequencies became <0.01, and the potential scale reduction factor^78^ 1.00 for all parameters. The runs were also visually checked for lack of trends in Tracer v1.6^79^. The first 25% of trees were discarded from each run as burn-in, and a 50% majority rule tree was constructed. Maximum likelihood (ML) analysis was conducted in RAxML v.8.2.x^80^ using the GTR + G model and rapid bootstrapping for 10,000 iterations. Clades with less than 85% Bayesian support and less than 75% ML support, were collapsed.

Maximum likelihood composite genetic distances^81^ based on the Tamura & Nei^82^ method were calculated between morphotypes and clades in MEGA v. 7.020^83^ for each gene separately and for the concatenated sequences, with gamma corrections as estimated by jModelTest. Maximum likelihood composite distances for all genes were preferred to the models of DNA evolution used in phylogeny construction, so as to minimize the effect of correction factors in the comparisons between *Encope*, *Mellita* and *Lanthonia*.

We estimated the time of splitting of clades in BEAST v.1.8.2^84^, using a relaxed log-normal clock with calibrations from fossil *Encope* (see below). An uncollapsed ML tree (which was compatible with the Bayesian tree) constructed in RAxML was used in BEAST as a starting tree. Operators causing topology searches (“SubtreeSlide”, “Narrow Exchange”, “Wide exchange”, “WilsonBalding”) were turned off to force BEAST to place time estimates on the nodes of the ML tree, but BEAST was allowed to estimate branch lengths. The appropriate model for each gene partition was entered as a prior, but the program was allowed to estimate parameters and substitution rates specific to each gene. Each fossil date was used as the offset of an exponential prior with a mean adjusted so that the upper 95% percentile extended to twice the age of the fossil. Trees were linked across genes, clocks were unlinked. Distributions of priors for mean rates (ucld.mean) of all genes were uniform, ranging from 0.001 to 1^100^. Distributions of priors for standard deviations of rates (ucld.stdev) were exponential with a mean of 0.333. Nine separate runs, each of 10^8^ steps and starting from different random seeds, were carried out, saving every 1000^th^ tree. The logs produced from all runs were combined in LogCombiner v.1.8.3, after removing the first 10% of the steps of each run, and viewed in Tracer v.1.6^79^ to verify that there were no trends and that all effective sample sizes (ESS) were >218. Trees were also combined in LogCombiner v.1.8.3 after removing the first 10% from each run. The resulting file was thinned out by taking every 10^th^ tree and entered in Tree Annotator v.1.8.3 to estimate age of each node.

We used the following fossil dates to calibrate the tree: The oldest species assigned by Durham^22^, to *Encope*, *E*. *michoacanensis* from the Ferrotepec Formation (Mexico eastern Pacific), dates from the late Early to Middle Miocene. Durham^22^ referred to affinities between *E*. *michoacanensis* and Panamic Miocene species. The oldest *Encope* from Panama (which is undescribed) occurs in the lowest Lower Gatun Formation at Sand Dollar Hill, but does not closely resemble any extant species. This formation was dated to 11.0 Ma by Smith & Jackson^85^. We therefore used 11 Ma as the minimum age for *Encope*. Well-dated fossils of extant *Encope* occur in Pliocene and Pleistocene deposits. These were used to constrain minimum ages of nodes leading to species of this genus. *Encope aberrans* has been recorded from the Late Pliocene (3.60-2.59 Ma) (Intracoastal Limestone of the Florida Peninsular [Florida Museum of Natural History (FLMNH)/University of Florida (UF) 202668 and 111402, Pliocene Liberty County Florida], *E*. *michelini* from the Middle Pleistocene (0.78-0.12 Ma) from St. Lucie County, Florida. (FLMNH/UF 156407 and 200441, Anastasia Formation, Late Pleistocene, St Lucie County), *E*. *emarginata* from the Late Pliocene (3.60-2.59 Ma) of the Caribbean and Venezuela^86^ and the Pleistocene of Brazil^87^. Fossil *E*. *galapagensis* from the limestone of Isla Santa Cruz [California Academy of Sciences, Geology (CASG) 98241-2] and Isla Baltra (CASG 98243) in the Galapagos Archipelago are morphologically identical to extant members of this species, and based on the stratigraphy of Hickman & Lipps^88^, date from 2.58-1.8 Ma in the Galesian Stage of the Pleistocene. Durham^89^ reported *E*. *californica*, and *E*. *micropora* from the Pleistocene of the Gulf of California. He described possible fossil representatives of *E*. *grandis* as ancestral species/subspecies, with *E*. *shepherdi* restricted to the Late Pliocene and *E*. *grandis inezana* restricted to the Pleistocene. Morphological differentiation between *E*. *grandis*, and *E*. *grandis inezana* are very slight, and relates to small differences in the size of the marginal notches, the size of the interambulacral lunule, and the concavity of the abactinal system. In our study such characters were observed to vary greatly in extant *E*. *grandis* and, thus, do not reliably differentiate these forms. We therefore used the Gelasian Stage of the Early Pleistocene for the minimum age of *E*. *grandis*. *Encope perspectiva* is recorded from the Pleistocene near Santa Cruz, Oaxaca, Mexico^22^. *Encope borealis* has been recorded (CASG 68681) from Pleistocene deposits between Puerto Peñasco and Punta Borrascosa, Mexico. For fossils, the age of which was recorded generally as the Pleistocene, we used the date of the start of the Middle Pleistocene, 0.78 Ma, as a minimum age. In summary, the offsets used in the BEAST analysis were as follows: *Encope*: 11 Ma, *E*. *emarginata:* 2.59 Ma, *E*. *aberrans:* 2.59 Ma, *E*. *borealis:* 0.78 Ma, *E*. *galapagensis:* 0.78 Ma, *E*. *grandis*: 0.78 Ma, *E*. *michelini*: 0.78 Ma, *E*. *micropora*: 0.78 Ma, *E*. *californica*: 0.78 Ma. We cross-validated the estimated dates of each node of the tree by carrying out eight separate runs of 10^9^ steps, each with one fossil date removed. In every other respect, these runs were identical with the ones including all fossil constraints.

### Equipment and settings

Images of sand dollars in Figs 1 and 2 were taken by S.E.C. using a Nikon D60 digital SLR camera and were edited in Affinity Photo V. 1.5.2. Brightness and contrast were unchanged. Scanning electron micrographs of spines in Fig. 1 were taken using a FEI Quanta 400 environmental scanning electron microscope.

### Data availability

The data generated and or analyzed during the current study are available in GenBank with the Accession numbers MF616941 - MF617108 for COI, MF617109 - MF617276 for ATPase-8, MF617277 - MF617444 for ATPase-6, MF617445 - MF617612 for 16S and MF617613 - MF617780 for 28S. The phylogenetic tree based on concatenated COI, ATPase-6, ATPase-8, 16S and 28S data reconstructed with MrBayes and RAxML has been submitted to TreeBASE and can be accessed at http://purl.org/phylo/treebase/phylows/study/TB2:S21395.

## Electronic supplementary material

Table S1.

Table S2.
